# MicroRNAs as Salivary Markers for Periodontal Diseases: A New Diagnostic Approach?

**DOI:** 10.1155/2016/1027525

**Published:** 2016-06-27

**Authors:** Gerhard Schmalz, Simin Li, Ralph Burkhardt, Sven Rinke, Felix Krause, Rainer Haak, Dirk Ziebolz

**Affiliations:** ^1^Department of Cariology, Endodontology and Periodontology, University of Leipzig, 04109 Leipzig, Germany; ^2^Institute of Laboratory Medicine, Clinical Chemistry, and Molecular Diagnostics, University of Leipzig, 04109 Leipzig, Germany; ^3^Department of Prosthodontics, University Medical Center Goettingen, Goettingen, Germany

## Abstract

The aim of this review is to discuss current findings regarding the roles of miRNAs in periodontal diseases and the potential use of saliva as a diagnostic medium for corresponding miRNA investigations. For periodontal disease, investigations have been restricted to tissue samples and five miRNAs, that is, miR-142-3p, miR-146a, miR-155, miR-203, and miR-223, were repeatedly validated in vivo and in vitro by different validation methods. Particularly noticeable are the small sample sizes, different internal controls, and different case definitions of periodontitis in in vivo studies. Beside of that, the validated miRNAs are associated with inflammation and therefore with various diseases. Furthermore, several studies successfully explored the use of salivary miRNA species for the diagnosis of oral cancer. Different cancer types were investigated and heterogeneous methodology was used; moreover, no overlap of results was found. In conclusion, five miRNAs have consistently been reported for periodontitis; however, their disease specificity, detectability, and expression in saliva and their importance as noninvasive markers are questionable. In principle, a salivary miRNA diagnostic method seems feasible. However, standardized criteria and protocols for preanalytics, measurements, and analysis should be established to obtain comparable results across different studies.

## 1. Introduction

MicroRNAs (miRNAs) are endogenous ~22 nt RNAs that play important regulatory roles in animals and plants by targeting mRNAs for cleavage or translational repression [1]. Many multifarious investigations regarding miRNAs have demonstrated their essential roles in physiological and pathological processes in the immune response [2]. Additionally, miRNAs likely contribute to development and progression of systemic diseases, such as cancer [3] and vascular disease [4]. Consequently, there is considerable interest in use of miRNAs as not only diagnostic markers but also potential therapeutic targets for various diseases [5]. In particular, the roles of miRNAs in oral cancer and precancer have been investigated. MiRNA expression appears to differ between healthy tissue and squamous cell carcinoma tissues of the oral cavity, which is discussed in an existing review [6]. Furthermore, some investigations have linked precancerous lesions and their risk of becoming malignant to changes in miRNA expression [7].

Furthermore, miRNAs are associated with bacterial infections [8] and, thus, are most likely associated with infectious diseases of oral cavity, for example, dental caries, endodontic infections, and periodontitis. Kim et al. (2015), however, already give an overview of current findings regarding dental disease, including periodontal disease and oral cancer. Kim et al. (2015) also mentioned limitations of current diagnostic and potential benefit of salivary miRNA diagnostic [9]. This is the reason to discuss the roles of miRNAs in periodontal diseases. As of now, only a few in vitro and in vivo investigations regarding these roles are available. A recent review article demonstrated potential roles of miRNAs during periodontal inflammation, showing the considerable role of them in periodontitis [10]. Thereby, different roles and mechanisms are shown and a very comprehensive overview on molecular pathways is given; however, methodology was only discussed between selected studies.

As mentioned above, an interesting approach is the analysis of miRNA species in saliva. High quality miRNAs were shown to be detectable in saliva [11], and the possibility of their usage in oral cancer detection appears to have a high potential for future diagnostics [12]. This noninvasive approach could be relevant for further diseases of the oral cavity. Nevertheless, the potential usage of miRNA as salivary markers for periodontal diseases was already not discussed yet. As shown for oral cancer, using miRNA for noninvasive diagnostics seems feasible [12] and could therefore serve as a salivary marker for periodontitis. The potential is high, so maybe recent indispensable invasive diagnostics or X-ray could become avoided by salivary miRNA diagnostic in the future and an early diagnosis might help to prevent bone and, in conclusion, tooth loss.

Nevertheless, despite of the huge potential, many different results are available, both for salivary oral cancer diagnostics and for analysis of periodontal tissue. Accordingly, it is questionable if the reasons are methodological differences between the studies or maybe if the usage of miRNAs in general is unsuitable.

Therefore, this review article shows current results of in vitro and in vivo studies regarding periodontal diseases as well as results of selected current studies dealing with salivary miRNAs for oral cancer detection. The aim was to detect promising future perspectives, but also current challenges regarding this issue. In particular, methodological aspects are focused to detect potential limitations of miRNAs as noninvasive markers for periodontitis.

## 2. Methods

### 2.1. Search Strategy

Our literature research was performed using the online database PubMed (http://www.ncbi.nlm.nih.gov/pubmed). To obtain a broad overview of the available theme-specific publications, we used different keywords for our search. We used “miRNA AND periodontitis” as keywords to obtain results for periodontal diseases. We used “miRNA AND saliva AND oral disease” and “miRNA AND saliva AND cancer” as keywords to obtain relevant articles for noninvasive possibilities, that is, the use of saliva to investigate miRNAs in diseases of the oral cavity. Then, the reference lists of relevant articles were searched for further results. To interpret the results, relevant publications were searched and analyzed critically. Neither a meta-analysis nor other statistical comparisons of existing data were performed.

### 2.2. Selection Criteria

Only current full-length articles in English language were included. The results were verified for their relevance. The following selection criteria were defined: miRNA investigations for in vitro and in vivo studies examining periodontal disease. Additionally, in vivo human investigations regarding salivary miRNA analysis in oral cancer and precancer were included.

### 2.3. Selection Process

Following the literature search, all references were limited to relevant publications. In vivo periodontal disease investigations that did not address periodontal tissue were excluded. Salivary diagnostic studies that did not explicitly concentrate on miRNAs were excluded, and only investigations for oral cancer and precancer were included.

## 3. Results

### 3.1. Periodontal Diseases

#### 3.1.1. In Vitro

Eight in vitro studies, which detected different miRNAs, were included for periodontal disease (Table 1). Nahid et al. (2011) examined the expression of cytokines associated with inflammation and differences in expression of cytokines and miRNA during infection with live and heat-killed bacteria in THP-1 monocytes. They demonstrated that expression of miR-146a is associated with infections caused by periodontal pathogenic bacteria in vitro [13]. Another study indicated that miR-146a is significantly overexpressed in THP-1 cells after stimulation with LPS from* P.g.* [14]. Furthermore, miR-146a was shown to be a negative regulator of TLR-NF*κ*B pathway in human periodontal ligament cells after* P. gingivalis* LPS stimulation, therefore being involved in inflammatory response [15]. Moreover, expression of miR-146a and miR-146b-5p in human gingival fibroblasts after stimulation with* P.g.* LPS was also investigated in a study by Xie et al. (2013). They demonstrated that expression of these miRNAs significantly increased upon LPS stimulation and concluded that miR-146 could work as a negative regulator of inflammation in periodontal disease [16]. Differential expression of miR-146a and miR-155 in dental pulp and in gingival and periodontal fibroblasts after stimulation with LPS from* Escherichia coli* was also reported. The increased expression of miR-146a was the highest in gingival fibroblasts and decreased expression of miR-155 was only significant in gingival fibroblasts [17]. Naqvi et al. (2014) showed different expressions of miR-146a. In this in vitro study THP-1 macrophages that were stimulated with LPS from* Aggregatibacter actinomycetemcomitans*,* P.g*., and also* P.g.* grown on cigarette smoke extract were examined. The different LPS caused both some identical and also varied expressions of miRNAs in human macrophages. Moreover, LPS from* P.g.* grown on cigarette smoke extract compared to LPS from unaffected* P.g.* caused differential miRNA expression, particularly that of miR-29b in human macrophages [18].

Results reported in other two studies were more heterogenous: Ouhara et al. (2014) demonstrated upregulated miR-584 in human gingival epithelial cells after stimulation with* P.g.* [19]. Furthermore, Moffat and Lamont (2011) focused on miR-203 expression in gingival epithelial cells stimulated with* P.g*. In this study, 14 miRNAs displayed significant changes in expression after exposure to* P.g.*; of these miRNAs, miR-203 was examined in detail and showed higher expression in tissues after* P.g.* stimulation [20].

#### 3.1.2. In Vivo Animal

Nahid et al. (2011) investigated expression of miR-146a, miR-132, and miR-155 in spleens and maxillae of ApoE^−/−^ mice after infection with periodontal pathogenic bacteria* P.g.*,* Treponema denticola* and* Tannerella forsythia*. MiR-146a primarily showed a significant increase in both maxillae and spleens, while miR-132 and miR-155 showed only minor changes [13].

#### 3.1.3. In Vivo Human

Seven in vivo studies investigating differential expression of miRNAs in human tissue samples were included (Table 1). In particular, Lee et al. (2011) compared the expression of inflammatory miRNAs in healthy and inflamed periodontal tissue. Microarray analysis indicated that expression of six miRNAs was upregulated more than eightfold and that expression of 22 miRNAs increased more than fourfold in periodontal disease tissue compared with healthy tissue. Of these miRNAs, six could be validated by qRT-PCR [21]. A similar investigation considered miRNA profiles of healthy and inflamed gingival tissues. Microarray analysis of healthy and inflamed gingival tissues indicated that expression of five miRNAs was upregulated more than fivefold, 85 miRNAs, twofold to fivefold. Moreover, expression of 34 miRNAs was downregulated twofold to fivefold in inflamed tissue. 12 miRNAs were validated by qRT-PCR [22]. A study of Japanese patients identified 17 upregulated and 22 downregulated miRNAs; six were selected for validation [23]. A further investigation also detected differential miRNA expression by microarray analysis, wherein 91 upregulated and 68 downregulated miRNAs were found. Six miRNAs were validated, and potential target genes were investigated [24]. Furthermore, Motedayyen et al. (2015) showed a positive relationship between miR-146a and clinical parameters in patients with chronic periodontitis [25]. Thus, many miRNAs with differential expression as detected by microarray analysis have not been validated by qRT-PCR; these miRNAs will not be discussed (Table 1).

Additionally, two studies investigated the roles of miRNAs in periodontal disease in association with systemic factors and disease; thus, differential expression of miRNAs between obese periodontitis patients and nonobese periodontitis patients was examined. However, different, nonoverlapping miRNAs were reported in each investigation [26, 27].

Notably, criteria for patient selection and for classification of healthy and diseased patients were similar across all studies. However, differences exist between investigations in defining periodontal health and periodontitis (Table 2). Additionally the number of study participants for in vivo investigations varied enormously between studies with ten patients or less for each group in most cases (Table 1). Likewise, methodological approach was similar across studies: microarray based technology was often used to screen and identify miRNAs with differential expression, and qRT-PCR was used for subsequent validation of top-hits. However, detailed methods were different; thus, for example, different miRNA isolation methods were used. Also differences in statistical analysis are conspicuous, especially internal controls that distinguish between the studies (Table 1).

### 3.2. MiRNA Detection in Saliva as a Noninvasive Diagnostic Tool

Nine investigations were included that illustrate the potential of saliva as noninvasive diagnostic tool for miRNA analyzes. We focused on salivary miRNA species analysis as a diagnostic method for oral cancer and precancer detection, because no results of appropriate dental and periodontal disease investigations could be found. Six of included studies examined oral squamous cell carcinoma, one precancerous lesions, one esophageal cancer, and one tumor of parotid gland. Park et al. (2009) showed presence of miRNA species in whole saliva and in saliva supernatants and described differential expression of miR-125a and miR-200a between cases and controls. Besides that stability of exogenous (miR-124a) and endogenous (miR-191) miRNA in saliva was also examined in this study, and endogenous miRNA was shown to have higher stability than exogenous miRNA [28]. Another investigation found that miR-9, miR-191, and miR-134 appeared to be potential markers for noninvasive diagnosis of oral squamous cell carcinoma using saliva. Additionally, this study describes a reliable isolation method for miRNAs from even small volumes of saliva [29]. In a study by Hung et al. (2016) salivary miRNAs, specifically miR-31 and miR-21 were investigated in oral premalignant disorders, particularly showing miR-31 to be a sufficient marker for high risk disorders and malignant transformation [30]. Also Liu et al. (2012) examined miR-31, and its expression in saliva was compared with its expression in plasma; thereby, a correlation could be shown. These authors also observed a decrease in expression of miR-31 after surgical resection of primary tumor [31]. Similarly, Duz et al. (2016) reduced miR-139-5p expression in patients with tongue carcinoma and normalization after surgical treatment [32]. In a study investigating expression of salivary miRNAs in premalignant lesions of oral cavity, a correlation was found between deregulated miRNA expression in tissue and saliva, although miRNA concentrations in saliva were lower [33]. A further investigation compared expression of miRNA in saliva between patients with oral squamous cell carcinoma, patients with oral squamous cell carcinoma in remission, patients with oral lichen planus, and a healthy control group. In addition to differences in miRNA expression between the groups, miR-27b was significantly overexpressed in saliva of patients with oral squamous cell carcinoma. Furthermore, completely different profiles of miRNAs could be found in cancerous tissues compared to biological fluids [34]. Additionally, another study found miRNA-184 to have the potential to distinguish between OSCC and potentially malignant disorders [35].

In addition, altered miRNA expression in saliva was also verified for esophageal cancer [36] and parotid gland tumors [37]. Their methodologies are shown in Table 3. In principle, methods of measurement (microarray, qRT-PCR) are similar between the studies. Remarkable differences could be found regarding form of saliva, extraction methods, statistical analysis, and internal controls. The number of study participants was already higher than for periodontitis. However, heterogeneous groups sizes with partly seven or eight patients each group [33, 34] and partly 50 patients and more each group [28, 29] were investigated (Table 3).

## 4. Discussion

Several studies investigating putative role of miRNAs in periodontal diseases have been performed; however, spectrum of miRNAs identified was substantially heterogeneous. Both in vitro and in vivo investigations were performed for periodontal disease, and five miRNAs, that is, miR-142-3p, miR-146a, miR-155, miR-203, and miR-223, were validated in more than one study. Accordingly, we can conclude that these miRNAs may play important role and thus could become potential markers for periodontal disease. However, majority of identified miRNA species differed significantly across studies (Table 1).

**Figure 1:**
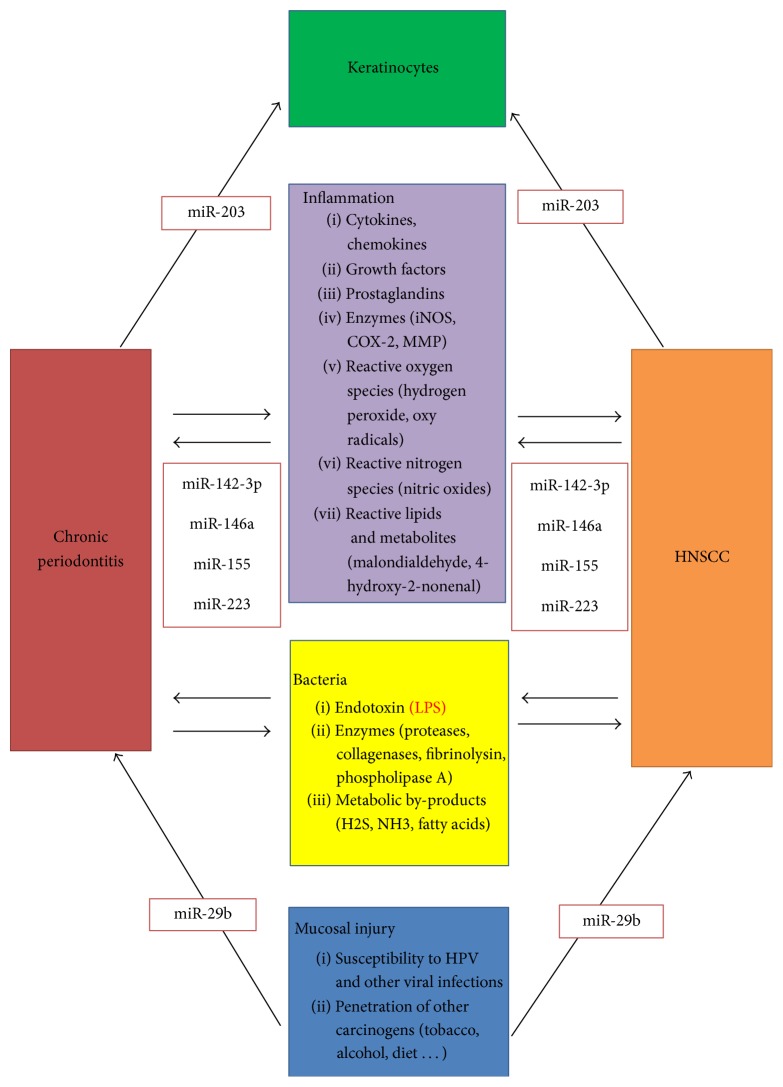
Model for the relationship among chronic periodontitis, HNSCC (modif. Han et al. 2014 [48]) and potential miRNAs that may be involved in the corresponding processes.

MiR-146a and miR-155 were dominant in in vitro studies. Otherwise, these miRNAs have also been identified in other inflammatory diseases and various cancers. MiR-146a seems to play a role in multifarious diseases and its function in bacterial infections has already been discussed [38]. MiR-155 has also been mentioned [39]. LPS may have a substantial effect on the expression of miR-146a and miR-155 [40]. Consequently, their altered expression during periodontal disease is not surprising because bacterial virulence factors such as LPS are relevant in pathogenesis of periodontitis [41]. These miRNAs are associated with many other inflammatory diseases, which might be indicative of a limited specificity. Additionally, miR-142-3p is associated with inflammation and LPS exposure [42, 43]. MiR-203 may also play an important role in inflammation and correlate with LPS exposure [44]. Interestingly, miR-203 expression is associated with immune reaction of skin [45, 46]. MiR-203 expression in keratinocytes is important; therefore, this miRNA may also be involved in gingivitis and periodontitis as determined by its effect on gingival keratinocytes. Finally, miR-223 also plays an important role in inflammation and LPS exposure [47]. Accordingly, correlations between these five miRNAs and periodontal disease are possible. Interestingly, these miRNAs appear to play a role in oral cancer. Thus, their roles in periodontal disease should be further examined (Table 4) as it might indicate a link between periodontitis and head and neck squamous cell carcinoma (HNSCC).  Figure 1 shows a summary of the interactions between periodontitis and HNSCC [48], and some potentially involved miRNAs. In this context, miR-29b, which showed an in vitro correlation with LPS from* P.g.* grown on cigarette smoke extract [18], has also been examined for oral cancer [49]. These findings raise the question if these miRNAs could serve as diagnostic markers for periodontal diseases or simply reflect an increased inflammatory state associated with various diseases.

Another problem is the great diversity of results. Although many miRNAs were found, convergence between the investigations, particularly in vivo, is small, resulting in conflicting conclusions. Reasons for differences between investigations of periodontal disease may differ. On the one hand, small variety in patient selection and different clinical procedures potentially causes these differences (Table 2). For future studies an evident graduation, for example, Page and Eke 2007, should be used for standardization [50]. On the other hand, variations in methods used for miRNA detection could play a crucial role (Table 3). A comparison of methods used suggests that miRNA detection procedures were quite similar. However, many differences in detailed procedures are evident. Using microarray and quantitative RT-PCR for analyzing and validating miRNAs are essential components of most investigations. However, detailed procedures for methods primarily differ, so several arrays from different manufacturing companies were performed. Importantly, differences in all of chosen criteria can be found between the investigations. Besides variations in statistical analyses it is important that internal controls differed in most cases.

Another aspect that could be crucial for the varying results is the small number of study participants. One study even investigated only three healthy and three diseased individuals [23]. A large group of 86 patients was selected only once [24]. The use of small sample sizes increases vulnerability to a range of errors and biases and may be a major reason for heterogeneous study results and potential false-positive findings. Similar issues were faced in early human genetic association studies, where many of reported genotype-phenotype associations could not be replicated in subsequent studies [51, 52]. Today, replication of initial findings in second cohort is considered essential for establishing the credibility of a genotype-phenotype association [53] and similar approaches could be adopted for studies reporting associations between miRNAs and diseases. Clearly, complex reason for differences in results exists. Different methods, clinical criteria, small groups of patients, and small specificity of miRNAs for periodontal disease could be causal complex.

In summary, comparability of results for the roles of miRNAs in periodontal disease is questionable. Potential candidate miRNAs as promising markers of periodontal disease do not appear to be specific for periodontitis.

Use of salivary miRNAs as noninvasive diagnostic markers has been studied in context of oral cancer, precancer, esophageal cancer, and parotid gland cancer so far. Again, reported results of these studies were quite divergent with little overlap in identified miRNA species. Importantly, these investigations used similar methods for miRNA detection in periodontal tissue and saliva; however, detailed methods are quite different (Tables 1 and 3).

In principle, detectability of miRNAs in saliva seems certainly possible, so, for example, a current study was able to detect noncoding RNAs in saliva: 127 to 418 miRNAs could be detected in each sample of human cell free saliva, with miR-223-3p being most abundant [54]. Interestingly, miR-223 is also found in periodontal tissue. Different studies have examined saliva to identify miRNAs as potential markers for oral cancer and precancer and report remarkably diverse results (Table 3). Strangely, basic principles of investigations were similar, but different internal controls, methods for miRNA isolation, statistical analysis, and forms of saliva (stimulated/unstimulated, whole saliva/supernatant) were chosen. Notably, number of study participants is already higher in these investigations than in studies of periodontitis, but also heterogeneous with partly small group sizes (Tables 1 and 3). In addition, form of saliva may play an important role, especially whether stimulated or unstimulated saliva is used may be relevant as well as exact procedure for obtaining saliva and criteria for patient selection. Furthermore, lack of stability of exogenous miRNAs in saliva may result in quick changes of miRNA concentrations from bacteria and inflammatory reactions [28]. Potential concentrations of miRNAs in body fluids in exosomes could also affect their detectability in human saliva samples [55]. Beside of that, however, it was mentioned that there are vesicle-free noncoding RNAs in saliva [54]. Furthermore, it is questionable whether identified miRNAs from tissue investigations are also potential markers for salivary diagnostic methods. The finding of completely different miRNA expression between cancerous tissue and body fluids [34] could be similar for periodontitis.

Detailed procedures of miRNA extraction and detection could also be relevant. In a recent study by Lundegard et al. (2015) expression of miR-203 was examined in whole saliva using two different PCR methods. The study concluded that detecting low levels of miRNA in saliva is difficult; more efficient extraction methods and more sensitive PCR techniques are necessary to use saliva as a reproducible source of miRNAs [56]. Because of differences in methods used, it is questionable if investigations are comparable at all. Some miRNAs may have variable expression between different microarray, PCR, and preparation methods [57]. Reproducibility and standardization of procedure appear relevant. Validated protocols such as those already described [58] may help to standardize the procedures. This would allow better reproducibility and provide more meaningful and more comparable results. This is supported by results of Hung et al. and Liu et al. both showing miR-31 to be a potential marker for malignant tumor [30, 31]. As shown in Table 3, both studies used same form of saliva, extraction, and validation methods as well as internal controls (Table 3). Accordingly, use of saliva for diagnosis of periodontal disease under condition of uniform methods seems conceivable.

However, the mechanism of miRNA infiltration in saliva during periodontal diseases must be considered. Thereby, exosomes might play a key role in miRNA transport from different cells into saliva [59]. In periodontal tissue, the junctional epithelial layers might be of highest relevance in this context. The junctional epithelium contains only few desmosomes and has therefore widened intercellular spaces [60]. Accordingly, this epithelium allows transport of several molecules between tissue and gingival crevicular fluid (GCF) [61]. Additionally, an enlarged permeability with an increase of GCF flow is observed during gingival and periodontal inflammation [61]. Consequently, high amounts of miRNA might pass the junctional epithelium, arriving GCF and thus saliva.

If comparable results can be achieved, further investigations will be of interest. For example, changes in miRNA expression after surgical resection of a tumor, as already described [3, 32], could become clinically useful by demonstrating improved periodontal conditions after therapy as well. Therefore, it could be assessed whether periodontitis therapy was successful by analyzing expression of specific miRNAs. Additionally, correlation between periodontitis and systemic diseases could be illustrated by altered miRNA expression [26, 27]. Although this topic seems to have great potential to provide further insights regarding oral disease, a critical view is needed. Currently, we have insufficient knowledge regarding oral disease and the roles of miRNAs in pathological processes. At present it is impossible to provide a clear statement regarding real relevance and possibilities of miRNA analysis. Nevertheless, using miRNAs to understand oral diseases, particularly periodontitis, and potential use of miRNAs as noninvasive markers or as therapeutic targets could be a great approach, which justifies basic research in any case. In conclusion, we can confirm unequivocally that salivary miRNA diagnosis for periodontal disease is a revolutionary idea. However, considering that additional investigations and standardized methods are required, possibilities of exploiting potential could be estimated only in the future.

## 5. Conclusion

Besides similar methods regarding miRNA extraction, profiling, and validation, there are methodical differences between studies, especially in internal controls and sample size, resulting in heterogeneous results. In principle, salivary miRNA diagnostic methods seem feasible. However, in our opinion, standardized criteria and protocols should be established and followed exactly to obtain comparable results. Five miRNAs related to inflammation are available, which may be used as potential markers for periodontitis. However, their detectability and expression in saliva and, accordingly, their importance as noninvasive markers are questionable.

## Figures and Tables

**Table 1:** Comparison of methods from current investigations regarding miRNAs in periodontal disease.

Author and year	Object of investigation	RNA extraction method	Detection method for miRNA profile	Validation of specific miRNAs	Number of study participants	Internal control	Statistical analysis	Investigated and/or deregulated miRNAs^*∗*^
In vitro human								

Moffatt and Lamont 2011 [20]	Gingiva epithelial cells	TRIzol (Invitrogen), miRNeasy kit (Qiagen)	Microarray (LC Sciences)	qRT-PCR TaqMan (Applied Biosystems)	—	RNU-48	ANOVA, *t*-test	**miR-203**

Nahid et al. 2011 [13]	Human THP-1 monocytes	mirVana miRNA isolation kit (Ambion)	—	TaqMan microRNA assay (Applied Biosystems)	—	RNU44	One-way analysis of variance followed by the two-sided, unpaired Student's *t*-test	**miR-146a** **miR-155** miR-132

Honda et al. 2012 [14]	THP-1 cells and THP-1-derived macrophages	TRIzol reagent (Invitrogen)	Agilent human miRNA microarrays (Release 12.0) (Agilent technologies)	TaqMan microRNA Assay (Applied Biosystems)	—	RNU44	Unpaired *t*-test, ANOVA-Williams test	**miR-146a** **miR-155**

Xie et al. 2013 [16]	Human gingival fibroblasts	TRIzol reagent (Invitrogen)	miRNA microarray analyses (Kangchen Bio-Tech)	qRT-PCR analysis (SYBR Green qPCR Master Mix PA-112, SABiosciences, Qiagen)	—	U6 small nuclear RNA	Student's *t*-test	**miR-146a** miR-146b-5p

Sipert et al. 2014 [17]	Human fibroblasts from dental pulps, gingivae, and periodontal ligaments	TRIzol (Invitrogen)	NCode miRNA rapid labeling system (Cat. # MIRLSRPD-20) (Invitrogen) Ncode Multi-Species miRNA microarray kit V2, (Invitrogen)	Taqman miRNA assays, (Applied Biosystems)	—	U6B	Two-way ANOVA followed by Bonferroni post hoc test	**miR-146a** **miR-155**

Naqvi et al. 2014 [18]	Human THP-1-differentiated macrophages	miRNeasy kit (Qiagen)	NanoString nCounter miRNA assay (NanoString Technologies)	Quantitative real-time PCR EvaGreen Master Mix (Biotium)	—	RNU6B	Student's *t*-test (two-tailed)	miR-29b miR-32 **miR-146a** miR-891

Ouhara et al. 2014 [19]	Simian virus 40 antigen immortalized gingival epithelial cell line, OBA-9	mirVana miRNA Isolation Kit (Applied Biosystem)	miRCURY LNA microRNA Array, v.16.0 (Exiqo)	TaqMan MicroRNA Assays System (Applied Biosystems)	—	RNU48	Student's *t*-test, Tukey honestly significant difference	miR-584

Jiang et al. 2015 [15]	Human periodontal ligament cells	TRIzol reagent (Invitrogen)	—	Quantitative RT-PCR analysis (RT SYBR® Green qPCR Master Mixes PA-112, SABiosciences, Qiagen)	—	U6 small nuclear RNA	ANOVA and Student-Newman-Keuls test	**miR-146a**

In vivo animal								

Nahid et al. 2011 [13]	Maxillae and spleens from ApoE^−/−^ mice	mirVana miRNA isolation kit (Ambion)	—	TaqMan microRNA assay (Applied Biosystems)	—	snoRNU202	One-way analysis of variance followed by the two-sided, unpaired Student's *t*-test	**miR-146a** **miR-155** miR-132

In vivo human								

Lee et al. 2011 [21]	Gingival tissue	mirVana*™* miRNA Isolation kit (Ambion)	RT^2^ miRNA PCR array system (SABiosciences)	TaqMan miRNA assays (Applied Biosystems)	n.i.	RNU44	Student's *t*-test	miR-181b miR-19b miR-23a miR-30a miR-let7a miR-301a

Xie et al. 2011 [22]	Gingival tissue	TRIzol reagent (Invitrogen)	miRNA microarray (Kangchen Bio-Tech)	Quantitative RT-PCR analysis (RT SYBR Green qPCR Master Mixes PA-112, SABiosciences, Qiagen)	10 periodontitis patients 10 healthy subjects	U6 small nuclear RNA	Unpaired Student's *t*-test	miR-126 miR-20a miR-142-3p miR-19a let-7f **miR-203** miR-17 **miR-223** miR-146b **miR-146a** **miR-155** miR-205

Stoecklin-Wasmer et al. 2012 [24]	gingival tissue	TRIzol (Invitrogen) RNeasy (Qiagen,)^*∗*^	Microarray^*∗∗*^	qRT-PCR	198 gingival tissue samples, 158 diseased and 40 healthy samples from 86 patients with periodontitis	n.i.	R (the R Development Core Team, 2005) and bioconductor statistical frameworks	miR-451 **miR-223** miR-486-5p miR-1246 miR-1260 miR-141

Perri et al. 2012 [26]	Gingival tissue	TissueLyser LT and miRNeasy Mini Kit (Qiagen)	Quantitative microRNA PCR array miRNA PCR Array (SABiosciences)	—	10 nonobese patients and 10 obese patients each group with 5 periodontally healthy sites and 5 chronic periodontitis sites	SNORD48 and RNU6-2	ANOVA and nonpaired Student's *t*-test	**miR-142-3p** miR-15a miR-30e miR-30d miR-22 miR-130a miR-106b miR-103 miR-185 miR-210 miR-18a

Ogata et al. 2014 [23]	Gingival tissue	miRNeasy Mini Kit (Qiagen)	Human miRNA microarray 8 × 15K kit (Agilent Technologies)	Real-time PCR SYBR Advantage qPCR Premix (Clontec)	3 chronic periodontitis patients and 3 edentulous residual ridges	U6	One-way ANOVA	miR-150 miR-200b **miR-223** miR-144 miR-379 miR-222

Kalea et al. 2015 [27]	Gingival tissue	mirVana miRNA Isolation Kit (Ambion)	Affymetrix GeneChip miRNA 3.0 arrays (Affymetrix)	TaqMan MicroRNA expression assays (Applied Biosystems)	36 eligible individuals	U6snoRNA and RNU6B	Partek 6.6 and 1-way analysis of variance, R-statistical environment	miR-4721 miR-557 miR-196a miR-323a-3p miR-200b-5p miR-188-5p

Motedayyen et al. 2015 [25]	Gingival tissue	mirVana miRNA isolation kit (Ambion)	—	Real-time PCR (TaqMan Universal Master Mix II, no UNG, and hsa-miRNA146a kits, Applied Biosystems)	10 healthy controls, 20 chronic periodontitis patients	n.i.	Student's *t*-test or Mann-Whitney *U* test; Pearson test or Spearman test	**miR-146a**

RNA extraction and isolation methods are listed. The proof of quality and quantity and quality assurance are shown as important quality criteria to draw conclusions regarding the reproducibility and standardization of the investigations. Furthermore, methods for miRNA profiling and miRNA validation are listed. In addition, the number of study participants is shown to draw conclusions regarding the validity of the results. n.i.: no information, ^*∗*^only miRNAs which were validated by RT-PCR were considered, and ^*∗∗*^Stoecklin-Wasmer et al. refer to an earlier report by Demmer et al. 2008 [62]. MiRNAs which were reported in more than one study are highlighted in bold type.

**Table 2:** Criteria for patient selection.

Author and year	PD		CAL		BOP		Radiographic bone loss	
	Healthy	Periodontitis	Healthy	Periodontitis	Healthy	Periodontitis	Healthy	Periodontitis
Stoecklin-Wasmer et al. 2012 [24]	≤4 mm	>4 mm	≤2 mm	≥3 mm	negative	positive	n.i.	n.i.
Xie et al. 2011 [22]	<3 mm	≥5 mm	<1 mm	≥3 mm	n.i.	*∗*	No	Yes
Lee et al. 2011 [21]	≤3 mm	>5 mm	none	>3 mm	*∗∗*	n.i.	No	Yes
Perri et al. 2012 [26]	≤4 mm	>5 mm	n.i.	n.i.	Negative	Positive	No	Yes
Ogata et al. 2014 [23]	n.i.	≥6 mm	n.i.	>6 mm	n.i.	Positive	n.i.	n.i.
Motedayyen et al. 2015 [25]	<3 mm	n.i.	<3 mm	n.i.	n.i.	n.i.	No	n.i.
Kalea et al. 2015 [27]	n.i.	>5 mm	n.i.	*∗∗∗*	n.i.	n.i.	n.i.	*∗∗∗*

PD: pocket depth; CAL: clinical attachment loss; BOP: bleeding on probing; n.i.: no information. ^*∗*^GI > 1; ^*∗∗*^BOP in whole gingiva < 10%, ^*∗∗∗*^bone loss > 30%.

**Table 3:** Comparison of methods for salivary miRNA diagnosis.

Author and year	Number of study participants	Form of saliva	miRNA extraction method	Detection of miRNA profile	Validation	Internal control	Statistical analysis	Potential marker for carcinoma
Park et al. 2009 [28]	50 oral squamous cell carcinoma (OSCC) patients and 50 healthy matched control subjects	Unstimulated whole saliva and saliva supernatant	mirVana*™* miRNA Isolation Kit (Ambion) DNA-free*™* (Ambion)	RT-preamp-qPCR (Applied Biosystems)	(i) RT-preamp-qPCR (Applied Biosystems) (ii) Four-plex RT-preamp-qPCR for miR-142-3p, miR-200a, miR-125a, and miR-93.	U6 snRNA	Mann-Whitney *U* test	miR-125a miR-200a

Liu et al. 2012 [31]	45 patients with OSCC and 10 patient with oral verrucous leukoplakia 24 healthy individuals	Unstimulated saliva supernatant	mirVana PARIS Isolation kit (Ambion) DNase digestion	TaqMan miRNA assay system (Applied Biosystems)	TaqMan miRNA assay system (Applied Biosystems)	miR-16	Mann-Whitney test, Wilcoxon matched pairs test, and linear regression analysis, receiver-operating characteristics ROC analysis	miR-31

Yang et al. 2013 [33]	8 progressing LGD leukoplakias 7 nonprogressing LGD leukoplakias 7 healthy volunteers	Unstimulated saliva	RNeasy Micro Kit (Qiagen)	TaqMan® low density array (TLDA) qRT-PCR system (Applied Biosystems) were used for global miR expression analysis in tissue samples	TaqMan MicroRNA Assay (Applied Biosystems)	RNU6	Random variance *t*-test, Benjamini-Hochberg false discovery rate (FDR) method, Mann-Whitney *U* test, Student's *t*-tests (two-sided)	miR-10b, miR-145, miR-99b, miR-708, miR-181c, miR-30e, miR-660, miR-197

Xie et al. 2013 [36]	39 patients with esophageal cancer and 19 healthy controls	Stimulated whole saliva and supernatant	mirVana PARIS Kit (Ambion)	7 whole saliva samples for EC group and 3 for healthy group for Agilent microarray 11.0 (Agilent Technologies)	RT-qPCR SYBR Premix Ex Taq (TaKaRa)	miR-16	Mann-Whitney *U* test or the Kruskall-Wallis *H* test, *χ* ^2^ test, receiver-operating characteristics ROC curves, Spearman's correlation test	miR-10b, miR-144, miR-451 (in whole saliva) miR-10b^*∗*^, miR-144, miR-21, miR-451 (in saliva supernatant)

Matse et al. 2013 [37]	38 patients with malignant and 29 with benign parotid gland tumors	Whole saliva and supernatant	mirVana Paris kit (Ambion) DNase I (Qiagen)	TaqMan Human MicroRNA Cards (Applied Biosystems) TaqMan MicroRNA assays (Applied Biosystems)	TaqMan microRNA assays (Applied Biosystems)	U6 snRNA	Wilcoxon rank-sum test, ROC curves	hsa-miR-374, hsa-miR-222, hsa-miR-15b, hsa-let-7g, hsa-miR-132, mmu-miR-140-5p

Momen-Heravi et al. 2014 [34]	9 OSCC patients before treatment, 8 patients with OSCC in remission, 8 patients with OLP, and 9 healthy controls	Unstimulated whole saliva	RNeasy kit (Qiagen)	multiplexed NanoString nCounter miRNA expression assay (NanoString Technologies)	TaqMan MicroRNA assay (Applied Biosystems)	miRNA-191	1-way analysis of variance, followed by a 2-tailed Mann-Whitney *U* test or Student's *t*-test, ROC curves analysis	miR-27b

Salazar et al. 2014 [29]	5 HNSCC patients, 5 healthy controls for expression analysis 56 HNSCC patients, 56 healthy controls for validation	Unstimulated whole saliva	QIAzol lysis reagent (Qiagen) NucleoSpin miRNA kit (Macherey-Nagel)	miScript*™* miRNA PCR arrays (Qiagen)	RT-qPCR miScript SYBR green PCR master mix (Qiagen)	SNORD96A	Mann Withney *U*-test, ROC curves, Wilcoxon rank sum test, Bonferroni method	miR-9, miR-134, miR-191

Zahran et al. 2015 [35]	20 healthy controls, 40 potentially malignant disorders, 20 OSCC, 20 recurrent aphthous stomatitis	Unstimulated saliva supernatant	miRNeasy serum/plasma extraction kit (Qiagen)	—	RT-qPCR SYBR green PCR kit (Qiagen)	SNORD68	One-way ANOVA, *F*-test, Dunnett *t*-test, Scheffe's multiple comparison, two-tailed tests	miR-21, miR-184, miR-145

Duz et al. 2016 [32]	50 saliva samples from 25 TSCC patients (once prior to and once after surgical treatment)	Unstimulated saliva supernatant	mirVana PARIS kit (Ambion)	8 samples (4 TSCC patients 4 healthy control) using Agilent 8 × 60K human V19 miRNA microarrays	TaqMan MicroRNA assay (Applied Biosystems)	RNU6b	Two-sided Student's *t*-test, Receiver operating characteristic (ROC) curves, 95% confidence interval (CI)	miR-139-5p

Hung et al. 2016 [30]	20 patients with oral potentially malignant disorders, 24 healthy individuals	Unstimulated saliva supernatant	mirVana PARIS isolation kit (Ambion)	—	TaqMan microRNA assay (Applied Biosystems)	miR-16	Unpaired test, ROC curves, Kaplan-Meier method, log-rank test, Cox proportional hazard model	miR-21 miR-31

RNA extraction and isolation methods are listed. The proof of quality and quantity and quality assurance are shown as important quality criteria to draw conclusions regarding the reproducibility and standardization of the investigations. Furthermore, methods for miRNA profiling and miRNA validation are listed. In addition, the number of study participants is provided to draw conclusions regarding the validity of the results. The form of saliva is also listed.

**Table 4:** Comparison of miRNas frequently mentioned in connection with periodontal disease with results from selected oral cancer and precancer investigations.

	Kozaki et al. 2008 [63]	Park et al. 2009 [28]	Cervigne et al. 2009 [64]	Scapoli et al. 2010 [65]	Lajer et al. 2011 [66]	Lundegard et al. 2015 [56]
miR-146a	X		X	X	X	
miR-155			X	X	X	
miR-203	X					X
miR-142-3p		X	X			
miR-223			X		X	

Each miRNA that was validated in the context of periodontal disease was also mentioned in investigations for cancer and precancer of the oral cavity. This table provides a small exemplary overview. Consequently, even more studies with similar results are not shown here.
